# Physical Frailty among Urban-Living Community-Dwelling Older Adults in Malaysia

**DOI:** 10.3390/ijerph17186549

**Published:** 2020-09-09

**Authors:** Camilla Wahida Norazman, Siti Nur’Asyura Adznam, Rosita Jamaluddin

**Affiliations:** 1 Department of Nutrition and Dietetics, Faculty of Medicine and Health Sciences, Universiti Putra Malaysia, Serdang, Selangor 43400, Malaysia; camillawahida@gmail.com; 2 Malaysian Research Institute of Ageing, (MyAgeing) Universiti Putra Malaysia, Serdang, Selangor 43400, Malaysia; rositaj@upm.edu.my

**Keywords:** frailty, elderly, ageing, sarcopenia

## Abstract

Frailty is a multidimensional syndrome, which is a worldwide concern within the field of geriatrics due to the aggravating effect on the physical and mental functions of the elderly. This study aimed to determine the prevalence and risk factors of the frailty syndrome among urban-living community-dwelling elderly in Malaysia. A cross-sectional study was conducted among 301 community-dwelling elders. Frailty status was assessed using the Fried phenotype criteria. Depressive symptoms were assessed using the Geriatric Depression Scale (M-GDS-14), whereas the functional abilities and cognitive status were measured using the Lawton Instrumental Activities of Daily Living (Lawton IADL) scale and the Mini-Mental State Examination (MMSE-M), respectively. Malnutrition risk was observed through the abridged version (Short Form) of the Mini Nutritional Assessment (MNA-SF). Multinomial logistic regression analysis was employed to determine the significant predictors of the frailty syndrome. Three hundred and one elderly persons engaged in this study, with a mean age of 67.08 ± 5.536 ranging between 60 to 84 years old. The prevalence values of frailty and pre-frail were 15.9% and 72.8%, respectively, in which women appeared to be at a higher risk of frailty. The multivariate model revealed that frailty could be predicted from an increase in age, lower household income, being at risk of malnutrition, wasting (low skeletal muscle mass), and high serum C-reactive protein (CRP) level. A holistic approach is suggested for managing the frailty syndrome as it involves a decline in the multiple components of the geriatric syndrome.

## 1. Introduction

The expeditious rate of ageing in the global population has an obscure impact on global growth and the health care system. The Malaysian population estimates data in 2019 revealed that 10.3% of the total population is composed of the older age group. The figure signified a substantial increase in the older population by 0.3% from the previous year [1]. In particular, by the year 2040, Malaysia is forecasted to be an ageing country with an elderly population of about 14.5% [1] and the fourth-fastest ageing nation with an increase of 269% between 2008–2040 [2]. The paradigm shifts of decline in fertility and increased longevity are expected to play a significant role in the changes in the age structure. Consequently, this “greying” population will be presented with multiple comorbidities and a decline in health status, increasing the demand for health and social care well-being.

This fact represents a unique challenge in the public health care sector since the steady increase in the number of older adults means higher expenses in health care to compensate for the medical cost and expenditure for the elderly. Indeed, statistics have shown that an increment in the total dependency ratio is due to an increase in the old-age dependency ratio, almost a 3-fold increase from 7.4 (2010)–21.7 (2040) [3]. The total expenditure on health care was estimated to be RM57.36 million with 4.24% of gross domestic product (GDP). The health care cost per capita was reported to be RM1790 in 2017 [4]. While the data on the health care expenditure for older adults is not specified, a study has reported that in a year, overall, an average of about RM426.50 of costs was determined to be borne by the sampled elderly Malaysians in community dwellings [5].

Although, given the advancement of scientific and cultural aspects, people have argued about the benefits of extended life expectancy within a society, the public health system is still potentially at risk of unsustainability due to a parallel increase of age-related disabling conditions. One of these conditions is frailty, being the primary public health problem due to the harsh characteristics that can have an impact on the country’s socio-economy. These inevitable challenges in society, due to the transition trend towards an older population that demands better health care, long-term care, social care, and pensions, should be made known and addressed. The health status of older persons is generally depicted using measures of morbidity, mental health status, or disability. Due to the complex deterioration of health status experienced by older adults, the term frailty has been coined to describe the multidimensional concept. Commonly described as a physiological state of increased susceptibility to stressors due to a decline in multiple physiologic reserves and dysregulation [6], the definition of frailty remains elusive. Many geriatricians believe that frailty may have a fundamental biological basis and is a unique clinical syndrome of disability. Characteristics of frailty include an age-related decline in multiple physiological functions and an increase in vulnerability to adverse health outcomes, such as mortality, disability, institutionalization, and falls [6].

Fried and colleagues proposed a landmark presentation of the frailty phenotype concept, which was one of the most validated models of frailty [6]. The concept and definition of frailty were built on a broad consensus including the cycle of frailty to determine the biological syndromes using five criteria. The phenotype is defined as the presence of three of the five criteria of shrinking (unintentional weight loss), weakness (low handgrip strength), self-reported exhaustion, slowness (low gait speed), and low physical activity. The term pre-frail is used to describe the intermediate group, while the term robust describes individuals who do not possess any of the mentioned symptoms.

Frailty is a transient process. Until recently, the likelihood of transitions between different frailty states over time was still under discussion. As observed in a prospective cohort study, frailty among older persons is a dynamic process, characterized by frequent transitions between frailty states over time. Transitions to the undesirable state of frailty are more common than transitions to states of lesser frailty (robust). Although the probability of transitioning from being frail to non-frail is very low, even over an extended period of time, the possibilities of recovering from a frail state to a pre-frail and potentially to a non-frail state are still possible. Primarily, the likelihood of transitioning between frailty states was highly subjected to one’s preceding frailty state [7]. Much attention has been drawn to the fact that the intermediate state of pre-frail is a window of opportunity for reversing the frailty progression, which makes this state alluring for preventive strategies. Although extensive global studies have been conducted on frailty, figures among the Malaysian older population are still scarce. Moreover, the implementation of various tools to assess frailty has caused many challenges in determining the current state of the evidence. In-depth research and a holistic understanding of frailty provide new prospects for prevention, health promotion, and improved care for both population and clinical settings. This present study, therefore, aimed to address the prevalence of frailty among urban community elderly with a low socioeconomic background and the multiple components of the frailty syndrome, with the inclusion of biochemical and biomarker parameters in exploring frailty status [8].

## 2. Materials and Methods

A cross-sectional study was conducted among the elderly who were residing in the People Housing Project (Projek Perumahan Rakyat (PPR) at Kuala Lumpur between October 2018–January 2019. The government introduced the PPR Program as the resettlement of squatters to meet the needs of the low-income population [9]. Hence, PPR was chosen as the study location because of the poor and ageing population living there [10,11]. The lists of PPR flats were obtained from the Community Development and Urban Wellbeing Department at Kuala Lumpur City Hall (DBKL). Multistage random sampling was employed in the selection of study sites and subjects. Ten PPRs were randomly chosen, and the elderly who fulfilled the criteria were selected for the study. Older adult is defined as those aged 60 years or above, the cut-off age being adopted by the United Nations (UN) extracted from the Malaysian dataset [12]. The inclusion criteria of the study population were community-dwelling elderly aged 60 years old or above, healthy with no known existing terminal illness, able to ambulate without personal assistance, and a permanent residence in the study area for at least a year. The exclusion criteria included the presence of severe sensory deficits for locomotion, communication, drawing and writing; having unstable medical conditions; and the presence of terminal illness. This study was conducted in accordance with the guidelines established by the Ethics Committee involving Human Subjects (JKEUPM) from Universiti Putra Malaysia. A letter of approval for conducting this study at PPR Kuala Lumpur was granted by the Community Development and Urban Wellbeing Department, Kuala Lumpur City Hall (DBKL). Three hundred and ten older adults who were eligible for this study were recruited, but nine were excluded due to missing data. An interview-based survey was conducted by trained researchers at the community center of each of the PPR flats. In the case where a respondent was unable to respond to the interviewer due to a language barrier, the primary caregiver was asked to be a proxy respondent. An informed consent form was disseminated to the eligible subjects and signed to confirm their participation. Subjects received explanations about the benefits and possible risks of the study and that any participants who wished to drop out from this study would not be subjected to any penalties. Information from the subjects was to remain confidential and used for the sole purpose of this study. Subjects who agreed to participate were invited to the allocated session and were reminded a day before to fast overnight for 10–12 h.

### 2.1. Measurement of the Frailty Syndrome

The frailty syndrome was defined based on the well-established and standardized phenotype of frailty proposed by Fried [6]. The Fried criteria evaluated five phenotypes of the frailty syndrome, which denoted one point for each criterion met. The phenotypic interest criteria were shrinking, exhaustion, weakness, slowness, and low physical activity. Subjects who met zero criteria were defined as non-frail, while those who met one or two criteria were defined as intermediate, or pre-frail. Subjects who met at least three criteria were considered as being frail. A Malay language version of the protocol for Frailty Assessment Components: Standardized Protocols was used. Modifications were made for the cut-off points and the assessment of physical activity, to better fit the population. Physical activities of the subjects were assessed using the Physical Activity Scale for the Elderly (PASE) instead of the Minnesota Leisure Activity Questionnaire (MLAQ), which was originally proposed by the Fried criteria. The use of the Malay version of the PASE questionnaire was more applicable than the MLAQ as it has been validated among Malaysian elderly [13]. The PASE comprised 10 questions that quantified six “leisure time activity”, three “household activity”, and one “work-related activity” to assess the frequency, duration, and intensity of various activities done in the past seven days. The questionnaire took about five minutes to complete, and the scores were computed through the New England Research Institute (NERI) scoring manual, which was adjusted by gender. Subjects were identified as having low physical activity if the score fell in the lowest quartile of the Physical Activity Scale for the Elderly (PASE), which has been validated among the Malaysian elderly population.

### 2.2. Anthropometry Measurements

The anthropometric measurements included in this study were weight, height, and body mass index (BMI). The stature height of the subjects was measured using a portable stadiometer (SECA- PorTable 213, Seca, Birmingham, UK). The circumferences of the crucial body parts were also considered in the study, including waist circumference (WC), mid-upper arm circumference (MUAC), and calf circumference (CC). Body compositions that were assessed in this study were total body fat percentage and skeletal muscle mass using the OMRON Body Fat Analyzer (BIA; Omron Healthcare Co., HBF-375 prototype, Kyoto, Japan). Prior to the measurement procedures, all anthropometric instruments were calibrated weekly to ensure reproducibility of anthropometric readings. All measurements were taken twice based on their standard technique.

### 2.3. Measurement of Other Covariates

The validated Malay version of the Geriatric Depression Scale (M-GDS-14) was applied in the present study to determine the depressive symptoms among older adults [14]. The M-GDS-14 was validated among Malaysian elderly with the newly formed scale at a satisfactory level of Cronbach’s alpha coefficient of 0.84 and concurrent validity with the Montgomery-Asberg Depression Rating Scale MADRS (Spearman’s rho of 0.68) [14]. The M-GDS-14 contained 14 items, with a score of 5 or more to be considered as an individual having depressive symptoms.

The validated Lawton Instrumental Activities of Daily Living (IADL)-Malay Version (Lawton IADL-MV) was used to assess functional ability within this study [15]. Lawton IADL assessed the instrumental activities of daily living, which require greater complexity of the neurophysical functions. A subject was classified to have normal functionality when all eight (8) activities could be performed independently.

This study also employed the validated Malay language version of the Mini-Mental State Examination (M-MMSE-S) to assess cognitive function [16]. The M-MMSE was a valid and reliable screening tool for cognitive impairment in the Malaysian elderly population, and more applicable in the local context because of the predominant Malay language used compared to the original MMSE version. A systematic review database was suggested for the education aspect in MMSE to be adjusted for ensuring better screening since the performance of the MMSE has been widely reported to be highly dependent on age and education, literacy, ethnicity, and culture [17]. Thus, a cut-off point of 19, which was proposed in the M-MSE-S, was applied in this study [16].

A validated Mini Nutritional Assessment-Short Form (MNA-SF) was used in this study to assess the risk of malnutrition among the elderly [18] and was validated in a healthy older population. The MNA-SF consisted of six screening questions related to loss of appetite, unplanned weight loss, or acute diseases in the preceding three months, mobility, presence of depression or dementia, and BMI. A score of 7 or less was classified as malnourished, whereas a score of 8–11 was at risk of malnutrition. A score of 12 or above was considered as well-nourished [19].

### 2.4. Statistical Analysis

Data from the survey were analyzed using Statistical Package for Social Science (SPSS) Version 22.0 (IBM Corp, Armonk, NY, USA). Before the analysis, data were explored for normal distribution. The continuous data were presented in mean and standard deviation, whereas the categorical data were presented in cross-tabulation of frequency (n) and percentage. Descriptive bivariate analysis was used to compare the mean difference or proportion using the chi-Squared test of association and the Pearson product–moment correlation. Further analysis using multivariate analysis was done to test the predictive power of a set of variables using multinomial logistic regression. The dependent variable tested for this present study was divided into robust, pre-frail, and frail. All significant predictors in the bivariate analysis were added into the regression model to assess the predictive ability. All variables were checked for assumptions to justify the use of logistic regression for prediction purposes. The robust group was set as the reference category, and the alpha value was set at *p* < 0.05 as an indication where the difference was statistically significant.

## 3. Results

### 3.1. Sociodemographic Characteristics

Three hundred and one older adults were selected in this study with a mean age of 67.08 ± 5.536 years, ranging from 60–84 years old. The majority of the subjects were female (69.4%) and Malay (70.8%) (Table 1). More than half of the female older subjects were either single, divorced, or widowed. The majority of the subjects obtained a formal education, and only 10.6% did not receive a formal education. Based on the Malaysia income classification, most of the subjects were from the lower-income category of poverty (≤RM970) with a median monthly income of RM 800 (approximately USD186.92).

Half of the subjects were found to be physically dependent (51.5%), and 15% of them were cognitively declining. The prevalence of subjects having depressive symptoms was 25.9%, which was more apparent among female subjects. More than half of the subjects were reported to be overweight (39.2%) and obese (25.9%) with a mean BMI of 27.52 ± 5.48 kgm^−2^. Subjects had high fasting blood glucose (37.5%) and total cholesterol (76.7%) during the data collection.

### 3.2. Factors Associated with the Frailty Syndrome

Table 2 presents the factors associated with the frailty syndrome. The prevalence values of frailty and pre-frail from the study population were 15.9% and 72.8%, respectively, with women being at higher risk of frailty. This study did not find a significant association between gender and frailty risk. Those who were single and obtained non-formal education with lower monthly income were significantly related to frailty (*p* < 0.05). The elderly who were diagnosed with at least one chronic disease were at higher risk of being frail. Nonetheless, functional disability and cognitive impairment showed significant association with frailty, but not for depressive symptoms. From the anthropometric and body measurement perspective, BMI and calf circumference were reported to be significantly correlated with frailty. On the other hand, only hemoglobin and C-reactive protein (CRP) serum concentration appeared to be significantly related to frailty (*p* < 0.05) within the biochemical parameters.

### 3.3. Predictors of the Frailty Syndrome

The multivariate model (Table 3) revealed that frailty may be predicted by increasing age (OR = 1.319, CI: 1.157–1.505), low household income (OR = 0.999, CI: 0.998–1.000), being at risk of malnutrition (OR = 4.551, CI: 1.425–14.532), wasting (low skeletal muscle mass) (OR = 1.439, CI: 1.185–1.748), and high serum CRP level (OR = 1.159, CI: 0.205–0.981). Notably, malnutrition appeared to be the strongest predictor among all in predicting the frailty syndrome.

## 4. Discussion

The present study identifies 15.9% of the study respondents aged 60 years or above, living in the urban-poverty community as frail, with at least three phenotypes of frailty. The prevalence is remarkably higher when compared to previous local findings conducted in an urban setting [20]. By looking into the trajectory years, the frailty prevalence in Malaysia has since increased from 5.7% in 2015 [20], 8.9% in the following year [21], to 15.9% in 2019 as presented in this study. Moreover, frailty status in the rural setting is significantly higher (18.3% frail and 41.6% pre-frail) [22,23]. However, a conclusion is difficult to be drawn based on the variance in the frailty tools employed in these studies. Overall, the prevalence of frailty among Malaysian elderly is found to be higher compared to the results of most other Asian studies, which employed the definition of 60 years old as the old-age group and used the Fried phenotype in determining the frailty syndrome. The rate of frailty in the neighboring country (Singapore) was found to be the lowest, namely 5.7%, with 40.1% as pre-frail [24]. In China, the reported prevalence was 7.0% and pre-frail was 51.2% [25]. Recent studies conducted in Indonesia showed a prevalence of frail and prefrail as 8.1% and 61.6%, respectively [26], whereas the prevalence is reported higher among Thailand’s elderly, namely 13.9% and 50.9%, respectively [27]. The prevalence values reported in these studies are the representative sample of the older adult population in the particular country, and such extensive data on the frailty rate in the representative sample of Malaysian older adults are still unexplored.

This study finds a higher prevalence rate of frailty among older women compared to men, which has been discussed previously [28]. The probabilities of an individual becoming frail increases with advancing age as ageing is associated with a gradual decline in physical functioning. The occurrence doubled among subjects aged 70 years or above, where being physically frail is at 25.6% compared to those aged 60–69 years old at only 12.3%. The upward curvature of ageing with frailty indicates the accelerating rate of accumulative age deficits [29,30]. As the accumulation of health deficits accelerates with advancing age, older persons are, therefore, generally at an increased risk of vulnerability to poor homeostatic adaptation from internal and external stressors, which can result in a greater risk of death [7].

After model adjustment, physical disability is no longer a significant predictor of the frailty syndrome. The interplay may partly explain the insignificant association between calf circumference, as proposed by Landi, who argues that physical function declines with calf circumference, which is plausibly related to the decline in proximal muscle mass of older adults [31]. However, in this study, although half of the elderly were self-reportedly to be physically disabled, the majority of them were at low risk of muscle wasting (94.4%), while frail older adults exhibited no mean difference of CC with pre-frail and robust elderly. Such an insignificant relationship between physical disability and frailty, therefore, might be accounted for by objective measurements of CC, based on the illustrations [31].

About a quarter of the subjects (25.9%) in this study were identified as having depressive symptoms. The prevalence rate is lower compared to the depression prevalence among Malaysian elderly, as reported previously (30.1%) [32]. This study finds a direct relationship where the frailty score increases when depressive symptoms increase. However, after being adjusted for the model, depressive symptoms are no longer significant in predicting frailty status, which is in line with the literature that has reported similar results [33,34]. Nonetheless, a growing number of studies are showing that there is a close relationship between both, with observed higher probabilities of depression in frail and pre-frail [35,36]. The insignificant relationship between depressive symptoms and frailty, as observed in this study, can be explained by the fact that a majority of the subjects who were depressed were not living with other people. Those elderly who live with others have a social advantage and are at lower risk of developing frailty as they are more likely to engage in a higher amount of physical activities and be taken care of by other family members.

The prevalence of poor cognitive function reported in this study is at 15.0%, and the incidence is higher among frail individuals. Although the present study did not demonstrate the significant contribution of poor cognitive function towards frailty, higher mean scores of cognitive impairment are observed in pre-frail and frail older adults. This study confirmed previous findings where frail elderly have a higher rate of cognitive impairment than those who are pre-frail and robust [37,38]. Cognitive impairment has been reported to occur concurrently with frailty, as well as to precede frailty [39]. In a study where frailty was able to predict the cognitive status of the elderly, poor cognition was associated with a 2.5-fold higher risk of being frail and a 2.2-fold higher risk of being pre-frail, when compared to the highest level of cognitive function [40]. Given that the mean age of the study subjects was relatively young and that the symptoms accelerated with age, the relationship between cognitive impairment with frailty is not strong enough to be established as a significant predictor.

The present study revealed malnutrition as the strongest predictor of the frailty syndrome. The proportion of individuals suffering from poor nutritional status increases gradually with growing levels of frailty. Winter and colleagues reported that one-third of those who were at risk of malnutrition were overweight or obese, indicating that elderly persons can be at risk of malnutrition despite being overweight or obese [41]. The study confirms the present finding where 51% individuals of overweight and obese were at risk of malnutrition compared to normal BMI (41%). The pathophysiology of malnutrition and frailty is known to share a common pathway. As discussed earlier, the Fried criteria and the components of MNA both are partly mirroring several components such as the assessment of immobility, exhaustion criteria as well as involuntary weight loss [42]. Although several components of Fried criteria and the MNA reflect a similar concept, each has its own distinct assessment approach, which makes them distinct from each other. Although still not fully understood, the overlapping pathway is apparent as they present similar phenotypes of weight loss and shrinking but may respond distinctively to treatments due to different etiologies [43].

The present study demonstrates that more than half of frail subjects (60.4%) were presented with a BMI of 30 kgm^−2^. The relationship between BMI and frailty is, therefore, positively correlated but does not appear to be a significant predictor in the model, which has been reported in other studies [44]. The paradox of obesity with mortality has been presented since 2005, where the antecedent shows a U-shaped association of body mass index with mortality risk [45]. Such an association has been observed in a large body of studies [45,46,47], with both ends of the weight continuum being of concern. Obesity needlessly protects an individual from frailty, or is often regarded as the wasting syndrome. Obese elderly can develop sarcopenic obesity and musculoskeletal frailty through a more complex mechanism, which is not evaluated in the assessment of BMI. Despite all these pieces of evidence, BMI has been regarded as an unreliable indicator of obesity, especially in older adults because of the incapacity of differentiating lean mass from adipose tissue. BMI also poorly represents central fat mass and nutrition, for which more reliable parameters should instead be used [48]. Thus, the interpretation of the current finding may be biased as BMI distribution is skewed towards the higher BMI extreme values, providing a disparity distribution of both lower and upper extreme values to establish the U-shaped relationship. As mentioned in a previous study, the small number of subjects can be a limiting aspect to achieve the statistically significant association of BMI with frailty [44]. Another study demonstrates a significant association between the skeletal muscle mass with frailty. A significant lower muscle mass among elderly observed in this study supports the proposed theory of sarcopenia being the main contributor of frailty [49]. As an individual ages, the decline in muscle mass and quality is inevitable; this leads to a progressive loss of muscle mass and strength [50] that is often treated as the primary component of sarcopenia. Sarcopenia is accompanied by risks of adverse outcomes [51], which can exacerbate the progression of frailty [52]. The clinical characteristic of frailty involves inadequate functional capacity, which is related to the number of skeletal muscles or functions [53]. As muscle strength is part of physical frailty components, both show a significant association with each other. However, the weakness component of the frailty syndrome merely relies on handgrip assessment. Handgrip strength has been shown to be a good predictor of mortality by providing affirmative estimations of total body muscle strength [54]. Hence, handgrip strength can be used to measure upper-body strength objectively, although it may not entirely reflect the body composition in lower extremities [55]. The present finding reiterates results from the broad literature of studies in demonstrating the significant association of skeletal muscle loss with frailty [55,56,57].

Among all the biochemical parameters tested in the present study, a significant association is only found between C-reactive protein among frail elderly. The significant association between CRP and frailty can be explained as the frail subjects in this study constitute overweight individuals. Pro-inflammatory cytokines such as C-reactive protein (CRP) and interleukin 6 (IL-6) have been introduced as the biomarkers of frailty, which is apparently higher among those who are overweight [58]. Pro-inflammatory cytokines may influence frailty either directly by promoting protein degradation, or indirectly by affecting critical metabolic pathways [59]. A meta-analysis study reported that both pre-frail and frail elderly have significantly higher CRP, Tumor Necrosis Alpha (TNFa)-R1, TNFa-R2, and IL-6 levels than the robust group [60]. In a previous study, an increment of CRP plasma concentration by 37% was also shown among pre-frail compared to frail elderly [61]. On the contrary, other studies have not found any significant differences in the concentration of C-reactive protein levels in pre-frail individuals [62,63]. Another study has reported that those classified as frail have significantly higher CRP, TNFa-R1, TNFa-R2, and IL-6 levels than those who are robust [64]. However, only TNFa-R1 and TNFa-R2 are found to be significantly associated with the pre-frail group [64], whose finding is similar to findings in this study where CRP is only related to the frail group but not with pre-frail elderly.

Several limitations should be acknowledged throughout the execution of this study. The nature of an observational study is limited to the interpretation and the cause–effect mechanism of the current findings. Furthermore, in this observational study, results may be confounded by other factors that are uncounted for in the study. It is essential to highlight that the study population generally was of relatively young older adults, with the majority of them aged between 60–69 years old. For this reason, the findings should not be generalized to all aged older adults and should be interpreted carefully. Hence, the association with predictors that are more prevalent with advancing age could not be established in this study.

This study presented the frailty status and associated factors among Malaysian community-dwelling older adults living in an urban setting with a low socioeconomic background. The present study also recognizes the frailty syndrome as a multidimensional construct that is not always straightforward. Further research in a more extensive set of the population is essential to substantiate the current findings, which advocate a holistic approach for managing the frailty syndrome, which involves a decline in the multiple components of the geriatric syndrome. Multidisciplinary engagement is, therefore, suggested in which the involvement of a medical practitioner, physiologist, nutritionist, and dietitian as well as a geriatric researcher are of importance in the identification and management of the frailty syndrome at the primary stage. Abdominal obesity and low skeletal muscle mass that appear to be significant contributors to physical frailty can be prevented by early engagement with physical and endurance activities. Therefore, education that focuses on the practicability of physical exercise for older adults should be integrated into intervention and health care programs to enhance the awareness of managing the frailty syndrome at a primary level.

## 5. Conclusions

Findings from this study reveal that frailty can be predicted by increasing age, low household income, being at risk of malnutrition, as well as having a low skeletal muscle mass and high serum CRP level. It is worthwhile to mention that quite a few predictors are considered modifiable factors, which highlights the importance of considering these modifiable factors in the intervention plan. A holistic approach is suggested for managing the frailty syndrome as it involves a decline in the multiple components of the geriatric syndrome. The implementation of a strategic plan is obviously necessary to reinvigorate those who are frail or pre-frail to the desirable robust state, as frailty is subject to change.

## Figures and Tables

**Table 1:** Sociodemographic characteristics of subjects (*n* = 301).

Characteristics	Total (*n* = 301)	Male (*n* = 92), 30.6%	Female (*n* = 209), 69.4%
Age (years)	67.1 ± 5.5	67.2 ± 5.5	67.0 ± 5.6
Ethnic groups			
Malay	213 (70.8)	65 (70.7)	148 (70.8)
Chinese	37 (12.3)	14 (15.2)	23 (11.0)
Indian	51 (16.9)	13 (14.1)	38 (18.2)
Marital status			
Single/Divorced/Widow/Widower	142 (47.2)	18 (19.6)	124 (59.3)
Married	159 (52.8)	74 (80.4)	85 (40.7)
Educational level			
No formal education	38 (12.6)	4 (4.3)	34 (16.3)
Formal education	263 (87.4)	88 (95.7)	175 (83.7)
Living arrangement			
Living alone	37 (12.3)	9 (9.8)	28 (13.4)
Living with others	264 (87.7)	83 (90.2)	181 (86.6)
Monthly household income (RM), mean (median, IQR)	959.9	1307.6	806.9
	(800.0, 700.0)	(1000.0, 1100.0)	(600.0, 650.0)
Monthly household income classification (RM)			
≤970	178 (59.1)	38 (41.3)	140 (67.0)
>970	123 (40.9)	54 (58.7)	69 (33.0)
Occupational status			
Not working/Retired	228 (75.7)	63 (68.5)	165 (78.9)
Still working	82 (27.2)	29 (31.5)	44 (21.1)
Functional ability			
Normal functional ability	146 (48.5)	55 (59.8)	91 (43.5)
Functional disability	155 (51.5)	37 (40.2)	118 (56.5)
Cognitive status			
Normal	256 (85.0)	87 (94.6)	169 (80.9)
Cognitive impairment	45 (15.0)	5 (5.4)	40 (19.1)
Depressive symptoms			
Normal	223 (74.1)	69 (75.0)	154 (73.7)
Depressive symptoms	78 (25.9)	23 (25.0)	55 (26.3)
Body mass index (kgm^−2^)	27.5 ± 5.5	26.2 ± 4.9	28.1 ± 5.6
Body part circumference (cm)			
Waist circumference	92.1 ± 13.4	92.4 ± 11.9	92.0 ± 14.0
Mid-upper arm circumference	29.7 ± 4.2	29.7 ± 4.1	29.7 ± 4.3
Calf circumference	34.3 ± 4.6	34.8 ± 4.3	34.1 ± 4.7
Body composition			
Body fat percentage (%)	36.1 ± 6.5	29.1 ± 5.1	39.2 ± 4.3
Skeletal muscle mass (kg)	28.1 ± 7.8	22.6 ± 4.9	30.5 ± 7.6
Skeletal muscle index (kgm^−2^)	12.0 ± 3.8	8.6 ± 2.0	13.5 ± 3.4
Biochemical parameters			
Hemoglobin	13.3 ± 1.5	14.2 ± 1.6	12.9 ± 1.2
Albumin	43.1 ± 2.1	43.0 ± 2.2	43.1 ± 2.1
Fasting blood glucose	6.9 ± 3.2	6.9 ± 3.6	6.8 ± 3.0
HbA1c	55.6 ± 22.5	56.8 ± 24.6	55.2 ± 21.5
Total cholesterol	5.3 ± 1.1	4.9 ± 1.1	5.5 ± 1.1
High Density Lipoprotein	1.4 ± 0.3	1.2 ± 0.2	1.5 ± 0.3
Low Density Lipoprotein	3.2 ± 1.1	3.1 ± 1.1	3.3 ± 1.1
Triglyceride	1.7 ± 0.9	1.8 ± 1.0	1.6 ± 0.8
C-reactive protein	4.8 ± 7.3	5.1 ± 10.8	4.6 ± 5.1

RM = Ringgit Malaysia, IQR = Interquartile Range.

**Table 2:** Factors associated with the frailty syndrome.

Characteristics	Robust (*n* = 34)	Pre-Frail (*n* = 219)	Frail (*n* = 48)	χ^2^/r	*p*-Value
Age (year)	63.9 ± 3.3	67.05 ± 5.3	69.4 ± 6.6	0.265 ^c^	0.000 ***
Age groups (year)				12.482 ^a^	0.002 **
60–69	31 (91.2)	161 (73.5)	27 (56.3)		
70 or above	3 (8.8)	58 (26.5)	21 (43.8)		
Gender				4.285 ^a^	0.117
Male	15 (44.1)	66 (30.1)	11 (22.9)		
Female	19 (55.9)	153 (69.9)	37 (77.1)		
Marital status				9.036 ^a^	0.011 *
Married	23 (67.6)	119 (54.3)	17 (35.4)		
Single/Divorced/Widowed	11 (32.4)	100 (45.7)	31 (64.6)		
Educational level				7.612 ^a^	0.022 *
No formal education	1 (2.9)	26 (11.9)	11 (22.9)		
Formal education	33 (97.1)	193 (88.1)	37 (77.1)		
Monthly household income (RM)	1366.5 ± 899.0	942.2 ± 764.0	678.1 ± 497.5	−0.250 ^c^	0.000 ***
Presence of chronic diseases				7.606	0.023 *
None	14 (41.2)	68 (31.1)	7 (14.6)		
≥1 chronic disease	20 (58.8)	151 (68.9)	41 (85.4)		
Functional status				11.821	0.003 **
Functional disability	13 (38.2)	107 (48.9)	35 (72.9)		
Normal functional ability	21 (61.8)	112 (51.1)	43 (27.1)		
Cognitive status				13.550	0.001 **
Normal	28 (82.4)	190 (86.6)	31 (64.6)		
Cognitive impairment	6 (17.6)	29 (13.2)	17 (35.4)		
Depressive symptoms				4.193	0.123
Normal	27 (79.4)	166 (75.8)	30 (62.5)		
Depressive symptoms	7 (20.6)	53 (24.2)	18 (37.5)		
BMI (kgm^−2^)				12.000 ^b^	0.006 **
Underweight	1 (2.9)	2 (0.9)	5 (10.4)		
Normal	14 (41.2)	69 (31.5)	14 (29.2)		
Overweight/Obesity	19 (55.9)	148 (67.6)	29 (60.4)		
Mid-upper arm circumference (cm)				7.824 ^b^	0.012 *
Normal	34 (100.0)	217 (99.1)	45 (93.8)		
Muscle wasting	0 (0.00)	2 (0.9)	3 (6.3)		
Biochemical parameters					
Hemoglobin	13.8 ± 1.5	13.2 ± 1.5	13.2 ± 1.3	−0.167	0.004 **
C-reactive protein	4.9 ± 6.2	3.3 ± 1.1	5.1 ± 7.9	0.090 ^b^	0.019 *

Note: ^a^ Chi-squared test of association. ^b^ Fisher–Freeman–Halton test. ^c^ Pearson product–moment correlation. * *p* < 0.05, ** *p* < 0.01, *** *p* < 0.001 indicate significant association.

**Table 3:** Predictors of the frailty syndrome.

Characteristics	Frail			Pre-Frail		
	OR	CI	OR	CI	OR	CI
**Sociodemographic factors**						
Age	1.319	(1.157–1.505)	0.000	1.214	(1.078–1.329)	0.001
Household income	0.999	(0.998–1.000)	0.029	1.000	(0.999–1.000)	0.441
Malnutrition						
Malnourished	4.551	(1.425–14.532)	0.011	1.733	(0.658–4.567)	0.226
Well-nourished	Reference					
**Anthropometric measurements**						
Waist circumference	1.042	(0.976–1.112)	0.218	1.007	(0.958–1.05)	0.797
Calf circumference	0.969	(0.839–1.131	0.691	1.006	(0.890–1.137)	0.797
Mid-upper Arm circumference	1.076	(0.888–1.304)	0.454	1.037	(0.898–1.198)	0.619
**Body composition**						
Body fat percentage	0.889	(0.803–1.007)	0.065	0.952	(0.878–1.033)	0.237
Low skeletal muscle mass	1.439	(1.185–1.748)	0.001	1.269	(1.089–1.478)	0.026
Visceral fat	1.016	(0.956–1.079)	0.646	1.016	(0.890–1.137)	0.922
**Blood parameters**						
Total cholesterol	1.461	(0.343–6.233)	0.608	0.449	(0.143– 1.406)	0.169
High-density lipoprotein	2.2568	(0.294–17.329)	0.434	3.300	(0.624–17.456)	0.160
Low-density lipoprotein	1.020	(0.980–1.020)	0.980	2.820	(0.836–9.509)	0.0.95
C-reactive protein	1.159	(0.205–0.981)	0.046	0.993	(0.476–2.072)	0.984

Note: Reference category: normal. OR: odds ratio; CI: confidence interval. The reference category is 1.00. Statistical significance at 0.05 level (two-tailed).
